# Temperature and Migration Intention: Evidence from the Unified National Graduate Entrance Examination in China

**DOI:** 10.3390/ijerph191610244

**Published:** 2022-08-18

**Authors:** Yan Chen, Xiaohong Chen, Hongshan Ai, Xiaoqing Tan

**Affiliations:** 1 School of Business, Central South University, Changsha 410083, China; 2 School of Frontier Crossover Studies, Hunan University of Technology and Business, Changsha 410205, China; 3 School of Economics and Trade, Hunan University, Changsha 410079, China

**Keywords:** climate change, temperature, highly educated talents, migration intention

## Abstract

This paper estimates the impact of destination cities’ temperature on the migration intentions of highly educated talents. Using a unique manually collected dataset of applicants for the Unified National Graduate Entrance Examination (UNGEE) of double first-class universities in China, we find that both hot (over 25 °C) and cold (below 5 °C) days in the previous 3 months before the registration date significantly decrease the number of applicants for the UNGEE of double first-class universities, relative to a moderate (20–25 °C) day. Heterogeneity analysis shows that such effects differ by destination universities’ quality and climate regions. We also find that destination cities’ income level can mitigate the negative effects of hot days and cold days on the number of applicants. These findings add to the existent literature by examining an understudied relationship between temperature and migration intention.

## 1. Introduction

Extreme temperatures, as a result of climate change, pose a fundamental threat to economic development and human welfare, especially in developing countries. A growing body of literature has examined the impact of temperature on economic and health outcomes: agricultural TFP and output [1,2,3], industrial output and productivity [4,5], employment [6,7,8], high-stakes cognitive performance and decisions [9,10,11,12], human capital [13,14], violence [15], time allocation [16], and physical and psychological health [17,18,19,20,21,22,23,24,25,26]. These studies find that extreme temperatures not only bring economic and social costs, but also impair human health. Some researchers have found that people may adopt avoidance strategies, such as relocation [27], internal migration [28], and international migration [29,30], to reduce extreme temperature exposure, especially in the most agriculture-dependent countries. However, there are insufficient studies drawing attention to the effects of temperature on migration intentions of highly educated talents, the cities’ core competence. Talent is becoming the core competitiveness of cities’ economic development both in developing and developed countries. As the foundation of human capital, talent is essential for innovation and sustainable development.

Previous studies have found that traditional economic factors including income [31], wage [32], labor market demand [33], and housing prices [34] affect individual migration decisions. Although a burgeoning literature focus on environmental factors, most of these studies examine the casual impact of origin cities’ and destination cities’ air pollution on college graduates’ job location choice [35,36], migration flows [37], short-term travel [38], settlement intentions [39], and emigration interests [40]. Relatively less attention has been drawn to the relationship between temperature and migration intentions.

In this paper, we study the impact of destination cities’ temperature on the migration intentions of highly educated talents using a unique manually collected dataset of applicants for the UNGEE, or kaoyan, of double first-class universities in China. The UNGEE is an examination organized by higher education institutions for the selection of graduate students in China, which is held annually. The UNGEE offers an opportunity to examine the effect of temperature on migration intentions for several reasons. First, the UNGEE has become a high-stakes exam for higher education institutions to select graduate students, which is held annually. The majority of applicants to double first-class universities are about to get or have already got a bachelor’s degree, which allows us to focus on highly educated talents. Second, applicants must choose a university to study for a master’s degree in the following two or three years when they register in the UNGEE. The university-city choice represents their intentions to move over at least the next few years. In addition, compared with the college-city choice after the National College Entrance Examination (gaokao), the colleges in which applicants pursue their masters’ degrees are more likely to be in cities where they want to work and live in the future. Third, the official registration period and preliminary examination date of the annual UNGEE is fixed, the former is from October 10th to 31st (9:00–22:00), the latter is on the penultimate weekend of December. Therefore, self-selection on the exam time due to temperature is impossible. In addition, most applicants for the UNGEE of the double first-class universities are fresh graduates and graduates with at least bachelor’s degrees. Therefore, they are highly educated talents.

Our main results show that hot and cold temperatures in the previous 3 months before the registration date significantly reduce the number of applicants for the UNGEE of double first-class universities, relative to moderate temperature. Compared with a day with daily mean temperature in 20–25 °C bin, an additional day over 30 °C, and below 5 °C significantly reduces the number of applicants for the UNGEE of double first-class universities by 108 and 302, respectively. There exists an inverted U-shaped relationship between temperature and the number of applicants. The decrease in the number of applicants in natural science due to hot days is larger than that in social science. However, cold days lead to a larger reduction in the number of applicants in social science. Our estimate is robust across alternative specifications, temperature measures, temperature bins, and samples.

In addition, we find heterogeneous impacts of temperature on the number of applicants of double first-class universities with different qualities. The negative impact of hot days is larger for first-class discipline universities and non-985 universities. Moreover, hot days increase the number of applicants for the universities located in cold regions, while cold days decrease the number of applicants for the universities located in hot regions. We also evaluate the nonlinear effects in different time spans. The results suggest that as the time span examined is closer to the registration start date, the negative effect of temperature becomes larger. Mitigation test indicates that destination cities’ income level is an important factor mitigating the negative effects of hot and temperatures on the number of applicants.

This study makes three contributions to the literature. First, to the best of our knowledge, this is the first paper to evaluate the causal impact of destination cities’ temperature on migration intention in China. There are few studies investigating the relationship between origin countries’ climate variability and international outmigration. Other researchers focus on the impact of climate change and climate amenities on internal migration in Brazil and household residential location choice in America. These studies mainly focus on population distribution and migration rate. However, the linkage between temperature and migration interest remains understudied. 

Second, this paper also contributes to the literature on the economic impacts of temperature. In addition to a large body of literature on the negative effects of temperature on economic output and health, some recent studies also find that extreme temperatures reduce cognitive performance and output, human capital, ability to work, and productivity. This paper adds to the literature by investigating the negative effects of temperature on migration intentions of highly educated talents. The cost of losing high human capital is becoming salient. Our findings provide implications for policymakers to focus on climate amenities to attract high human capital.

Third, this paper adds to the literature that explores the determinants of migration decisions and interests of highly educated talents. Elites with higher human capital respond more to pollution and climate amenities when they choose where to live, work, and study. Our conclusions confirm that temperature is an important factor for highly educated talents to consider when choosing where to go for further education.

The rest of the paper is organized as follows. Section 2 provides the empirical background of the UNGEE and double first-class universities in China. Section 3 and Section 4 present the empirical strategy and data. Section 5 provides the main results, robustness checks, and heterogeneous analysis. Section 6 discusses the effects of temperature on the number of applicants in different time spans and the mitigation effect. Section 7 concludes the paper.

## 2. Empirical Background

### 2.1. The Unified National Graduate Entrance Examination

According to the learning form, the type of postgraduate enrollment is divided into full-time and part-time graduate students. The part-time graduate students’ study time is flexible, usually on weekends. In addition, they do not have to stay in school every day. However, once the full-time graduate students are admitted, they must study for a master’s degree in school for the next two to three years. The universities, for which they choose to apply, represent their intentions to move over the next few years. Therefore, this paper focus on the number of full-time postgraduate applicants.

The UNGEE is divided into the preliminary examination and re-examination. Official registration period is from 10 to 31 October (9:00–22:00). The preliminary examination is held nationwide at the end of December every year, the penultimate weekend. There are four subjects in the preliminary examination, which forms the “2 + 1 + 1” subject system. “2” refers to two public subjects: political science and a foreign language usually English. The first “1” refers to one basic subject: mathematics or professional foundation. The second “1” refers to one specialized subject. The specialized subject examination paper is set independently by universities. Candidates are required to choose target universities in advance to review the exam subjects. Generally speaking, the period from the summer vacation to December is crucial for preparing for the UNGEE. The preliminary scores are released in March of the following year. Only when the score passes the national, university-level and school-level cut-off line, can applicants take the re-examination. The re-examination is independently organized by each school. According to the enrollment quota and the scores of the preliminary and reexamination, applicants are admitted by universities on the basis of competitive selection. Migration intention turns into actual migratory behavior.

### 2.2. First-Class Universities and Disciplines of the World

First-class universities and disciplines of the world, called “Double First-class”, is another national strategy following “Project 211” and “Project 985” in the field of higher education in China. In 2017, the Ministry of Education, the Ministry of Finance, and the National Development and Reform Commission jointly issued the list of first-class universities and disciplines of the world. In total, 42 universities have been selected for the country’s construction plan of world-class universities, and 95 universities have been selected for the country’s construction plan of first-class disciplines. Most double first-class universities are “Project 211” and “Project 985” universities, which are the top Tier 1 universities in China. The first-class universities’ comprehensive strength ranks ahead of all universities in China. The universities selected for the country’s construction plan of first-class disciplines have unique advantages and development prospects in certain disciplines. In addition, the central government has issued a series of policies to support the construction of double first-class universities. Double first-class universities are China’s elite universities. 

As the number of applicants increases dramatically, the difficulty of the UNGEE for these elite universities is greater than ordinary universities. According to the result of China’s seventh national census, the population with college education is 218.36 million, accounting for about 15 percent of the country’s total population. Most candidates who apply for postgraduates of the double first-class universities are fresh graduates and graduates with at least bachelor’s degrees. Therefore, applicants for the UNGEE for these elite universities are highly educated talents. Talent is the core competitiveness of cities’ economic development [41,42]. Therefore, the adverse effects of high and cold temperatures on the number of applicants for double first-class universities may further lead to talent loss and influence the competitiveness and development of cities.

## 3. Data

### 3.1. Data on Applicant for the UNGEE

**Figure 1:**
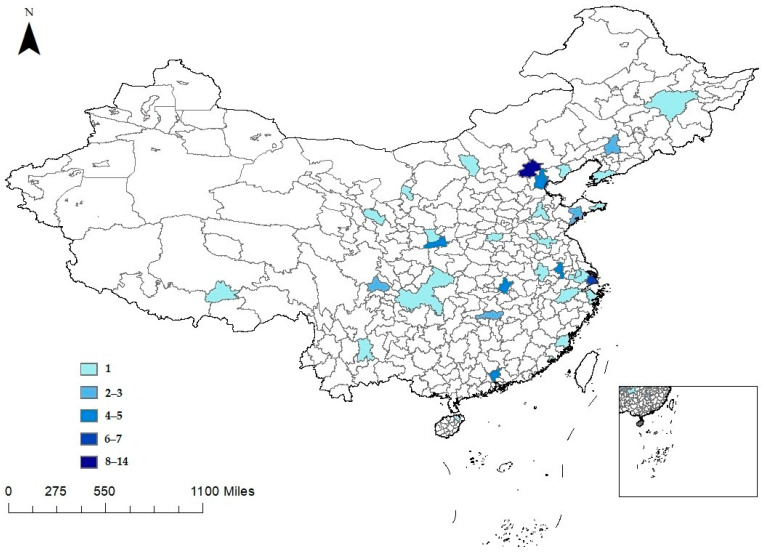
The geographical distribution of the number of double first-class universities.

We obtain the applicant data for the UNGEE from the official website of the graduate school of double first-class universities, which reports the number of applicants and admissions for each major of each school for the UNGEE, allowing us to explore the heterogeneity across different discipline categories (natural science versus social science). Appendix A shows further discussion of data source, which presents the process of collecting data manually. Because some universities do not issue the information on the applicants for the UNGEE on the official website, we only collect data on the applicants for the UNGEE from 75 double first-class universities, located in 34 cities, from 2010 to 2020. The double first-class universities included in our sample account for 54.745% of the total 137 double first-class universities. Due to the fact that some universities do not issue the information on the number of applicants in certain years, an unbalanced panel dataset includes 580 observations. Appendix B Table A1 presents the list of 75 double first-class universities. Figure 1 shows the distribution of the 75 double-first-class universities. 

### 3.2. Weather Data

Weather data includes daily average, maximum, and minimum temperatures, precipitation, average relative humidity, average wind speed, sunshine duration, and atmospheric pressure for 699 weather stations from 2009 to 2020, obtained from the China Meteorological Data Sharing Service System. We first use the inverse-distance weighting method to convert station data to city-level data. The distance from the city’s centroid to stations is controlled within 150 km. The closer the station is to the city’s centroid, the greater the weight will be assigned.

Applicants’ target universities are mostly chosen in advance to prepare the specialized subject exam. Generally speaking, the period from the summer vacation to December is crucial for preparing for the UNGEE. At the same time, most candidates’ university decisions are made about during this period. Therefore, we match the applicant data with destination cities’ weather data at that decision-making time (from July to October). First, we calculate the daily weather characteristics for each of the previous 90 days (3 months) before the registration start date (10 October). We next calculate the 90-day average mean value of each seven 5 °C temperature bins (>30 °C, 25–30 °C, 20–25 °C, 15–20 °C, 10–15 °C, 5–10 °C, <5 °C) to estimate the nonlinear effects of temperature on the number of applicants. To prevent multicollinearity, the 20–25 °C bin is defined as the baseline temperature bin, omitted in our regressions. The variables for the number of days in other six temperature bins are included in main regressions simultaneously. Therefore, we can estimate the nonlinear effects of temperatures on the number of applicants by comparing the average changes in applicants when one more day falls into one of six temperature bins relative to the baseline bin of 20–25 °C.

### 3.3. Summary Statistics

We first merge university-level data and city-level weather data based on city and registration start date. Table 1 provides summary statistics for all university-level variables and city-level variables. Panel A provides the summary statistics of applicants and admissions variables from 2010 to 2021. The number of applicants ranges from 237.00 to 41,522.00, with a mean value of 11,582.60. The average number of applicants in social science is more than that in natural science. Panel B provides the summary statistics of temperature variables. The average number of days with daily mean temperature in the seven bins (>30 °C, 25–30 °C, 20–25 °C, 15–20 °C, 10–15 °C, 5–10 °C, <5 °C) in the past 90-day period prior to registration start date is 7.24, 35.34, 28.70, 13.16, 4.87, 0.67, and 0.03, respectively. Panels C and D provide the summary statistics of other meteorological variables and air pollution variables. During our sample period, the mean PM_2.5_ concentration is 30.5, which is higher than the safe standard for human health recommended by the WHO. Panel E provides the summary statistics of annual city-level economic variables, used to examine the effectiveness of mitigation in Section 6.2.

**Figure 2:**
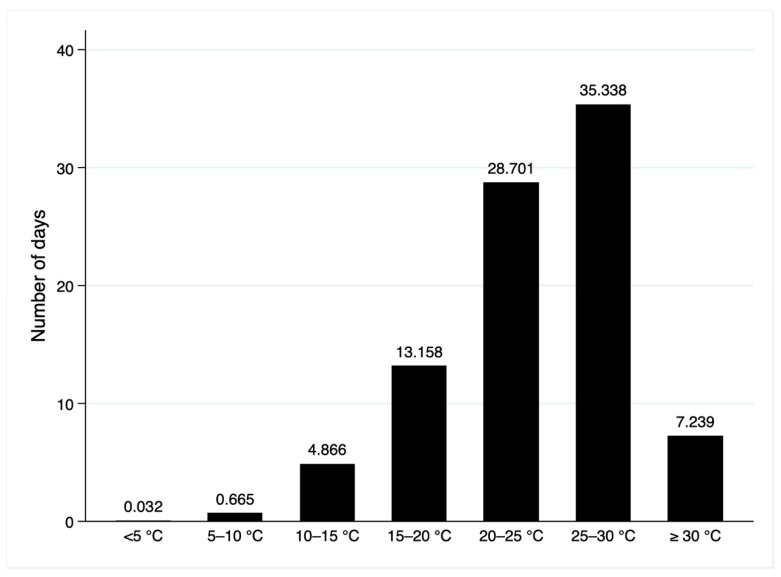
Distribution of daily average temperature in the previous three months in the estimation sample.

**Figure 3:**
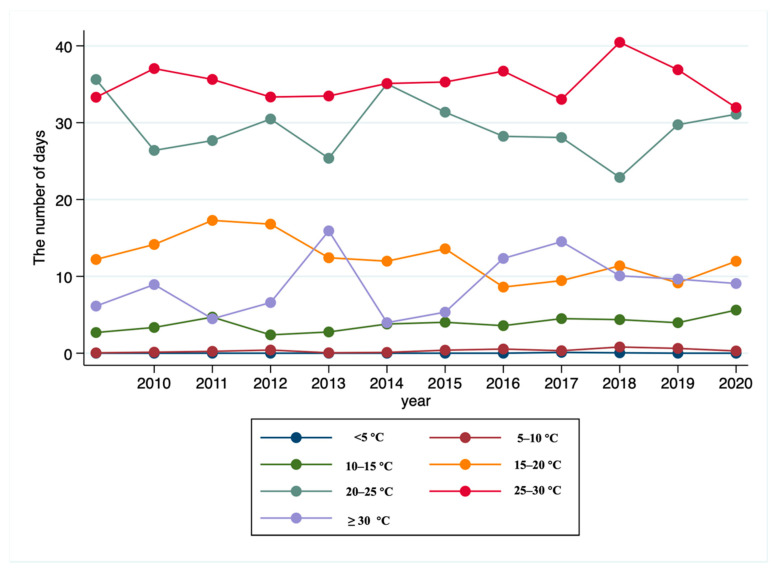
The annual trend of the number of days with daily average temperature falling into each temperature bin in the previous three months in the estimation sample.

**Figure 4:**
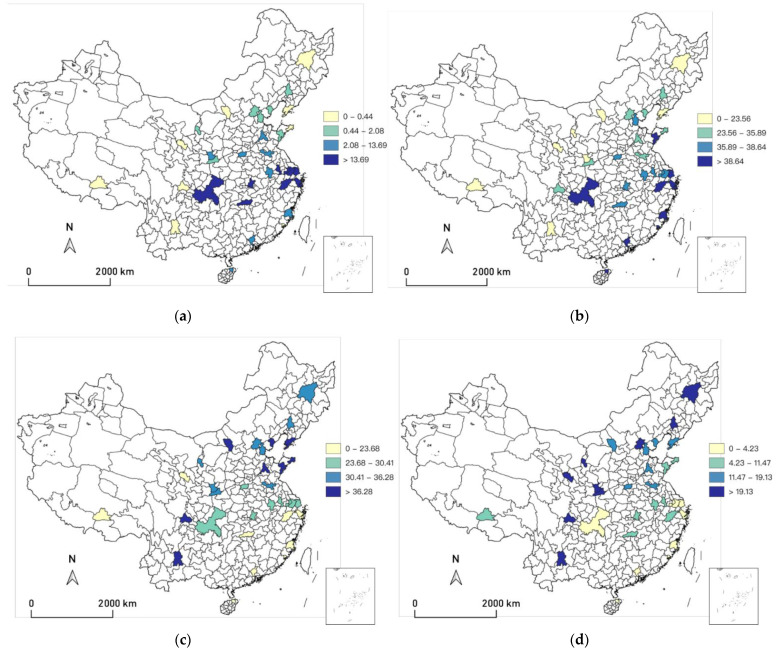

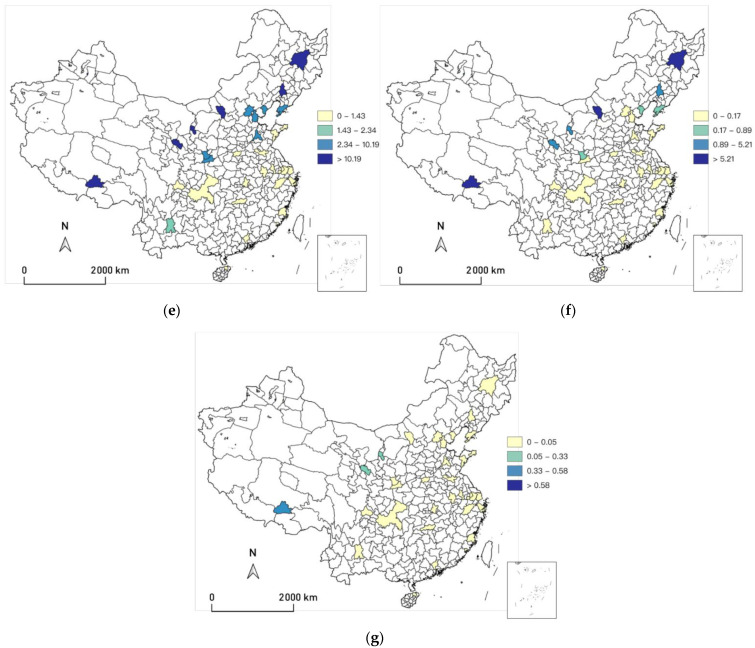
The geographical distribution of the number of days with daily average temperature in the previous three months in seven 5 °C temperature bins. (**a**) Number of days in the previous 90–day period with daily average temperature over 30 celcius degrees; (**b**) Number of days in the previous 90–day period with daily average temperature between 25 and 30 celcius degrees; (**c**) Number of days in the previous 90–day period with daily average temperature between 20 and 25 celcius degrees; (**d**) Number of days in the previous 90–day period with daily average temperature between 15 and 20 celcius degrees; (**e**) Number of days in the previous 90–day period with daily average temperature between 10 and 15 celcius degrees; (**f**) Number of days in the previous 90–day period with daily average temperature between 5 and 10 celcius degrees; (**g**) Number of days in the previous 90–day period with daily average temperature below 5 celcius degrees.

As shown in Table 1, Figure 2 depicts the distribution of the average number of days in seven temperature bins. Figure 3 plots the annual trend of the number of days with daily average temperature falling into each temperature bin in the previous three months. We find that the number of days with daily average temperature over 30 °C is generally on the rise from 2009 to 2020. The number of cold days (below 5 °C) does not change much. Figure 4 displays the geographic distribution of the average number of days in the seven temperature bins.

## 4. Empirical Strategy

The main objective of this study is to estimate the casual effects of temperature on migration intentions. We construct the following model to evaluate the nonlinear effects of temperature on the number of applicants based on the empirical specifications used by [19,23]:

(1) $$Y_{ict}=\beta_{0}+\sum_{j=1}^{7}\beta_{j}Temp_{c,t-1}^{j}+X_{ic,t-1}\gamma +W_{c,t-1}\theta +\mu_{i}+\pi_{ct}+\varepsilon_{ict}$$

where *I* denotes a double first-class university, *c* denotes the city in which the university locates, *t* denotes the year the university announced the number of applicants for the UNGEE. Registration generally started on 10 October of the previous year. $Y_{ict}$ is the number of applicants for the UNGEE of double first-class university *i* in city *c* in year *t*. The main variables of interest $Temp_{c,t-1}^{j}$ are the number of days with daily average temperature in the 5 °C bin *j* (from 1 to 7) in the 90-day period before the registration start date (10 October every year.) We use 20–25 °C temperature bin as the baseline group, in which people feel most comfortable. A vector of coefficients $\beta_{k}$ measure the average change in applicants when one more day falls into kth temperature bin relative to the baseline bin of 20–25 °C. $X_{ic,t-1}$ is a key university-level control variable, ratio of the number of applicants and the number of admissions. $W_{c,t-1}$ is a vector of weather variables, including precipitation, average relative humidity, average wind speed, sunshine duration, and atmospheric pressure. They are mean values of the 90-day period before the registration start date. 

We also control for university fixed effects ($\mu_{i}$) and city-by-year fixed effects ($\pi_{ct}$) University fixed effects absorb time invariant university characteristics (e.g., geographical location). City-by-year fixed effects control for time-variant city characteristics. We also conduct a robustness check using province-by-year fixed effects. $\varepsilon_{ict}$ is the error term. Because all weather variables are grouped at the city level, the standard errors may be biased downward (Moulton 1986). We cluster standard errors at city level, which allows for spatial autocorrelation and serial correlation within city over time.

## 5. Results

In this section, we first present the baseline results, where outcomes are defined as the total number of applicants, and the number of applicants in social science and natural science, respectively. Then, we report several robustness checks. Finally, we examine the heterogeneous effects of temperature on the number of applicants across different elite universities.

### 5.1. Main Results 

**Figure 5:**
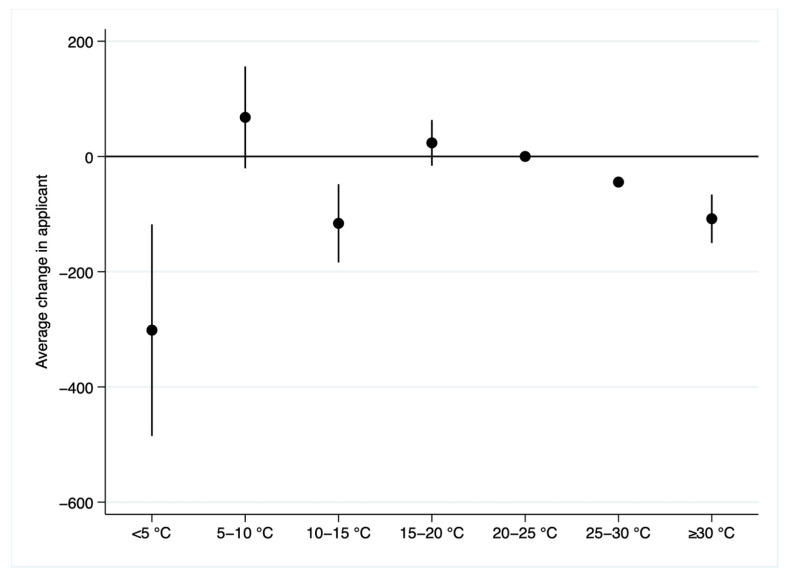
Estimated impacts of average temperature on the number of applicants for the UNGEE.

Table 2 presents the nonlinear effects of temperature on migration intentions. In column (1), we only include university fixed effects and year fixed effects. In column (2), we add university-level controls, the ratio of the number of applicants and the number of admissions. We further control for other weather meteorological variables in column (3). An additional cold day below 5 °C significantly decreases the number of applicants by 388, 386, and 432 in columns (1) to (3), respectively. Other temperature-bin day variables are insignificant across the three specifications. However, a large body of literature has found that destination city’s economic and population attributes and amenities are important factors in making location decisions. When these factors are omitted, the main results will be biased. Therefore, we replace city fixed effects and year fixed effects in column (3) by the city-by-year fixed effects in column (4) to control time-variant city-level variables. We find strong effects of hot and cold weather on migration intentions. The estimated results in columns 1 to 3 of Table 2 are biased because there are not control variables at city-year level. In column (5), we also include year fixed effects to test the robustness of the results. The estimated results are consistent with those in column (4). We use the results from column (4) for the interpretation of the remaining results. Relative to a day in 20–25 °C bin, an additional day with daily average temperature over 30 °C, in the 25–30 °C bin, in the 10–15 °C bin, and below 5 °C in the previous 3 months, significantly decreases the number of applicants by 108 (0.93% of sample mean), 44 (0.38% of sample mean), 116 (1.00% of sample mean), and 302 (2.60% of sample mean), respectively. Figure 5 plots the estimated coefficients and 95% confidence intervals. As noted earlier, the 20–25 °C bin is omitted as the reference group. There exists an inverted U-shaped relationship between temperature and the number of applicants. Although, compared with the coefficients on the high temperature bins, temperatures below 5 °C have larger negative effects on the number of applicants, the number of days with daily average temperature in the previous three months below 5 °C is much less than that over 30 °C. Back-of-the-envelope calculations indicate that the negative effect of hot days (108.094 × 7.239 = 782.49) is much larger than that of cold days (301.501 × 0.032 = 9.65).

**Figure 6:**
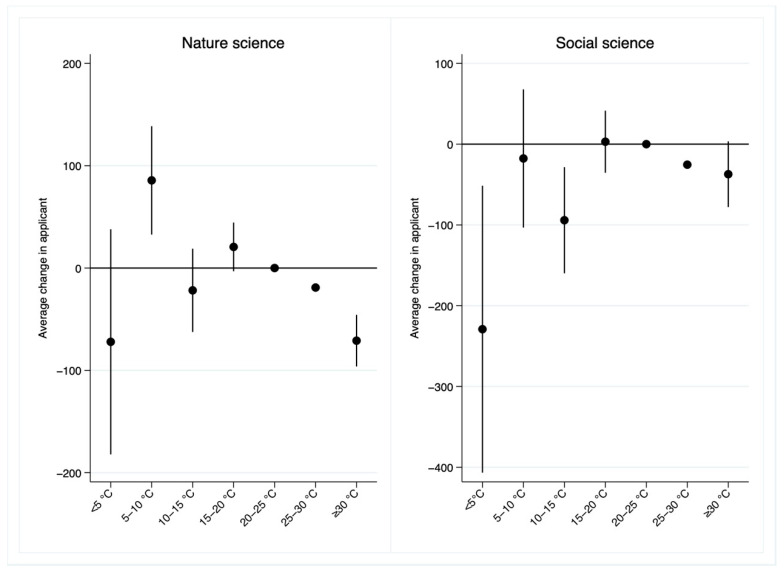
Estimated impacts of temperature on the number of applicants for the UNGEE by discipline category.

We further re-estimate the nonlinear effects of temperature on the number of applicants for different disciplines separately. Table 3 shows the main results. We find that the negative effect of high temperature is larger for the applicants in natural science and the negative effect of low temperature is larger for the applicants in social science. Relative to a day in 20–25 °C bin, a day over 30 °C significantly reduces the number of applicants in natural science by 71, which is about twice as large as the negative effect of high temperature on the number of applicants in social science. In addition, the effect of an additional day with daily average temperature below 5 °C significantly decreases 229 applicants in social science. The effect of cold days on the number of applicants in natural science is negative but insignificant. Figure 6 plots the estimated coefficients for the applicants in different disciplines.

### 5.2. Robustness Checks

We next conduct a battery of robustness checks to support our main results. First, we conduct various alternative specifications. Table 4 presents the results. Our baseline results use city-by-year fixed effects to control for time-variant city-level variables. In column (1), we replace city-by-year fixed effect by province-by-year fixed effects and city fixed effects. The negative effects of high temperature on the number of applicants remain significantly negative. Column (2) reports the results of a placebo test. We first calculate the number of days with daily average temperature in different temperature bins in three months after the registration end time. Then, we estimate their effects on the number of applicants. Our results show that all the estimated coefficients are insignificant, suggesting that unobservable confounding factors do not alter our main results.

Then, some studies have found that origin and destination cities’ air pollution can also impact people’s interest in migration [37,38,40]. We are concerned that city-by-year fixed effects cannot capture air pollution levels. Therefore, our results may be confounded by air pollution. We directly control air pollution levels by including the 90-day average PM_2.5_ concentration and examine the relationship between air pollution and migration intentions. City-level air pollution data from the Ministry of Ecology and Environment (MEE). The Chinese air quality real-time publishing platform, published by the China Environmental Monitoring General Station, is the most exhaustive source of Chinese air pollution data currently known that provides hourly data on atmospheric pollutants (including PM_2.5_, PM_10_, SO_2_, O_3_, CO, and the AQI; recorded once per hour) from more than 1000 different monitoring stations. In this paper, we focus on daily average PM_2.5_ concentration. The MEE only reports PM_2.5_ after 2014. Then, we calculate the 90-day average mean value of PM_2.5_ concentration. Furthermore, we examine the effectiveness of income in mitigating the effects of temperature. Table 5 reports the results. Column (1) is our baseline results from column (4) of Table 2. We re-estimate Equation (1) only for the sample of cities covered by PM_2.5_ from 2014 to 2020. Column (2) reports results. We add controls for air pollution in column (3). We find that the effect of temperature on the number of applicants remains unchanged, which suggests that air pollution do not confound our main results. We also find that air pollution significantly decreases the number of applicants for double first-class universities. Destination cities’ air pollution decreases highly educated talents’ interest in going to these cities to study for a master’s degree, which is consistent with previous findings [36,40]. 

**Figure 7:**
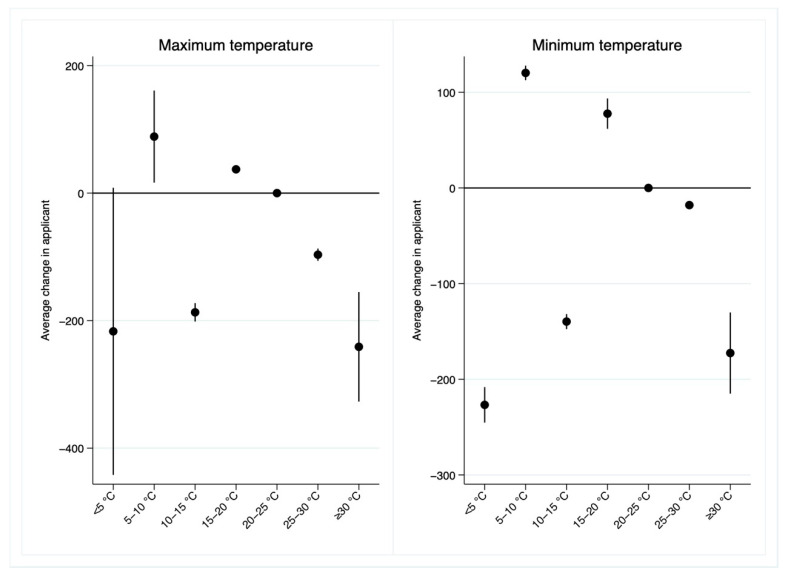
Estimated impacts of average temperature on the number of applicants for the UNGEE using alternative temperature measures.

Furthermore, we use daily maximum temperature and minimum temperature to construct the number of days following seven temperature bins in the 90-day period before the registration start date. We re-estimate Equation (1) to estimate the nonlinear effects of temperature on migration intentions. Results are shown in Table 6. Columns (1) and (2) report results by using temperature-bin variables with daily maximum and minimum temperature. Results are similar to the column (1). Figure 7 presents the nonlinear effects of daily maximum temperature and minimum temperature with the estimated coefficients from columns (1) and (2) of Table 6. 

**Figure 8:**
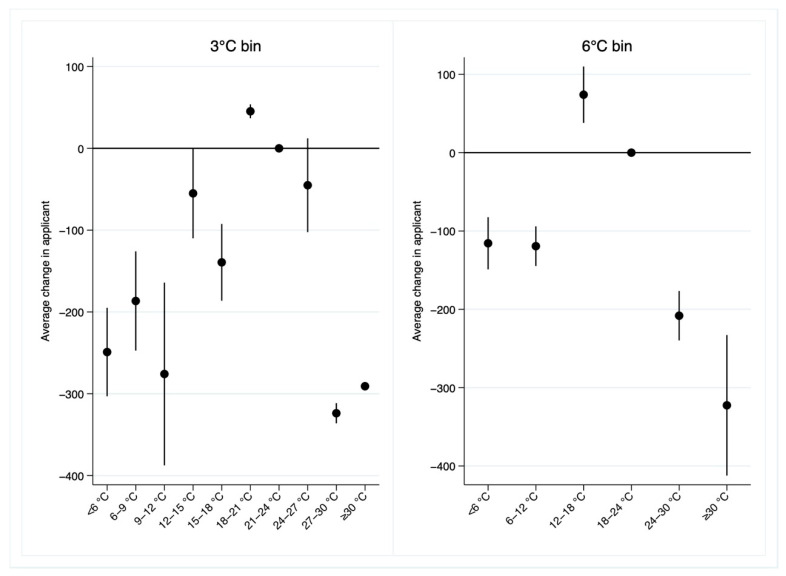
Estimated impacts of average temperature on the number of applicants for the UNGEE using alternative temperature bins.

In addition, we replace 5 °C bin with 3 °C bin and 6 °C bin and re-estimate Equation (1). Table 7 provides these results. Relative to a day in the 21–24 °C or 18–24 °C bin, an additional day in other temperature bins significantly decreases the number of applicants. These results are consistent with the main results in column (4) of Table 2. Figure 8 plots the estimated coefficients with 95% confidence intervals. The relationship between temperature and the number of applicants is approximately an inverted U-shape.

Finally, our main results may be biased due to inclusion of some special cities. We first exclude all observations in special cities and replicate the baseline regression. Beijing, Shanghai, Guangzhou, and Shenzhen, the top 4 developed cities in China, is more attractive to young people with extraordinary opportunities and benefits. In addition, Beijing, Nanjing, Shanghai, and Wuhan has more than 5 double first-class universities. Therefore, these cities attract more talents by virtue of the concentration of educational resources and opportunities. We exclude these cities, respectively. As shown in columns (1) and (2) in Table 8, we find that hot days still significantly reduce the number of applicants for elite universities. In column (3), we further include non-double first-class universities and re-estimate the main empirical model. We find that replacing a day with daily average temperature in the 20–25 °C bin with an additional day with daily average temperature in the other six temperature bins significantly reduces the number of applicants. However, the values of these estimated coefficients are smaller than the baseline results.

### 5.3. Heterogeneity Analysis

In this section, we explore possible heterogeneity in the effects of temperature on the number of applicants across universities’ type and tier and climate regions. 

First, we examine the heterogeneous effects of temperature across double first-class universities in different types. Comprehensive universities set up various disciplines and have a large number of admissions. These universities are characterized by elementary and multidisciplinary. In contrast, non-comprehensive universities, known as specialized universities, have distinct industrial backgrounds and outstanding advantages in industry-related disciplines (e.g., medicine, agriculture, forestry, politics and law). The discipline distribution of specialized universities is relatively concentrated. When a double first-class university is a comprehensive university, dummy equals to one, and otherwise equals to zero. As shown in column (1) of Table 9, we find that the effects of high and low temperatures on the number of applicants are homogenous across comprehensive and non-comprehensive universities.

We next examine the differential effects of temperature on the number of applicants for the UNGEE of double first-class universities in different country’s construction plan. Dummy equals to one if universities are selected for the country’s construction plan of word-class universities, and otherwise equals to zero. Results are presented in column (2) of Table 9. Relative to universities that have been selected for the country’s construction plan of first-class disciplines, the number of applicants for universities that have been selected for the country’s construction plan of world-class universities increases about 64 by an additional day with daily average temperature over 30 °C in the previous 90 days compared with a day in the 20–25 °C bin. 

Then, we compare the heterogeneous effects of temperature across universities in different tiers. A total of 985 universities are top elite universities in China, which have more advantages to attract applicants. Column (3) of Table 9 provides the estimated coefficients. Compared to their counterparts, hot temperatures and cold temperatures lead to more applicants to choose 985 universities to get master’s degree. These findings suggest that the tier of university may reduce the brain drain caused by temperatures.

Finally, we examine whether applicants’ university-city choices are affected by destination cities’ climate region. According to the Qinling Mountain-Huaihe River Line, we first divide the sample into northern universities and southern universities. The results are presented in column (4) of Table 9. The interaction of the seventh temperature bin and a dummy for northern cities is significantly negative. Relative to southern universities, an additional day with the daily average temperature below 5 °C decreases the number of applicants about 46 for northern universities, compared with the reference temperature bin. We further spilt the sample into cities that are below or above the median average 90-day temperature. Column 5 of Table 9 shows that hot weather increases the number of applicants for the universities located in cold regions, but cold weather decreases the number of applicants for the universities located in cold regions, relative to the universities located in hot regions. Our results suggest that destination cities’ climate conditions may influence talent loss. 

## 6. Discussion

In this section, we first discuss the effects of temperature on the number of applicants in different time spans of calculating the temperature-bin day variables. Then, we examine the mitigation effect of income on the effects of temperature on the number of applicants.

### 6.1. The Effects of Temperature on the Number of Applicants in Different Time Spans

Whether to take part in the UNGEE and which university to apply for are relatively long-term and complicated decisions. We examine the nonlinear effects of temperature on the number of applicants by calculating temperature-bin day variables in different time spans to comprehensively understand which period of temperature has the greatest effect on migration intentions of talents. Column (1) of Table 10 presents our baseline results in column (4) of Table 2. First, we estimate the effects of temperature on the number of applicants during the registration period (from 10 to 31 October). Results in column (2) of Table 10 show that all the coefficients on the number of days in temperature bins are insignificant, suggesting that temperature during the registration period does not have significant effect on the number of applicants. In general, the university, for which an applicant apply to study for a master’s degree, has been selected for before the registration date. Therefore, migration intentions should not be affected by the temperature during the registration period.

We then estimate whether the effects of temperature on the number of applicants differ by calculating the number of days in different temperature bins in the shorter time spans, including 1-month and 2-month period. Columns (3) and (4) of Table 10 provide the results. Relative to a day in the 20–25 °C bin, an additional day with the daily average temperature over 30 °C in the previous 30 days and 60 days before the registration date decreases the number of applicants by 170 and 118, respectively. An additional day with the daily average temperature in the 25–30 °C decreases the number of applicants by 48 and 38, respectively. Our results show that the closer the time span is to the registration date, the larger the impacts of high temperatures on the number of applicants become. In addition, replacing a day in the 20–25 °C with an additional day below 5 °C in the previous 30 days and 60 days can significantly decreases the number of applicants by 222 and 222, respectively, which is slightly smaller than the impact of low temperature in the previous 90 days before the registration date.

Finally, we further explore the effects of temperature over longer period on the number of applicants. We extend the time span to half a year and one year to calculate the number of days in different temperature bins. We provide the results in columns (5) and (6) of Table 10. We find that the coefficients of the number of days in high and low temperature bins become smaller. An additional day with daily average temperature over 30  °C and below 5 °C in the previous half a year significantly decreases the number of applicants by 47 and 167, respectively, compared with a day in the 20–25 °C. Replacing a day with daily average temperature in the 20–25 °C with a day over 30 °C and below 5 °C in the previous one year also reduces the number of applicants by 16 and 120, respectively. These findings imply that temperature is an important factor affecting migration intention in both the short and long period. However, the long-term impact is smaller than the short-term.

### 6.2. Tests for Mitigation

Then, we estimate the moderating effect of income on the impact of temperature on the migration intention. We use city-level per capita GDP and per capita wage as proxy variables of income, respectively, obtained from the China City Statistical Yearbooks. Then, we match the city-level income variables to university-level variables by city and year of registration. We next use the interactions of the number of days in each temperature bins and income examine how the extent of income could affect the effects of temperature on the number of applicants. Results are shown in Table 11. In column (1), except for the interaction of the number of days below 5 °C temperature bin and GDP per capita, other interactions are significantly positive. When GDP per capita increases ten thousand yuan, the number of applicants is 0.20%, 0.09%, 0.28%, 0.19%, and 0.07% less impaired by an additional day with the daily average temperature in the first (over 30 °C), second (25–30 °C), forth (15–20 °C), fifth (10–15 °C), and seventh (5–10 °C) temperature bins, respectively, relative to a day in the 20–25 °C temperature bin. However, the GDP per capita do not influence the relationship between the low temperature and the number of applicants. In column (2), all interactions are significantly positive. When annual average wage payment per capita increases ten thousand yuan, the number of applicants is less impaired by 0.41%, 0.09%, 0.10%, 0.35%, 0.09%, and 0.36%, by replacing a day with the daily average temperature in the 20–25 °C bin with an additional day in the first to seventh temperature bins, respectively. Our findings suggest that income can mitigate the negative effect of temperature on the number of applicants, especially for hot and cold temperatures. 

## 7. Conclusions

Climate change has become a major environmental factor affecting migration. In this paper, we study the impact of destination cities’ temperature on migration intentions of highly educated talents by using applicant data from 75 double first-class universities in China. We find that hot and cold days in the previous 3 months before the registration date significantly decrease the number of applicants for the UNGEE of double first-class university. There exists an inverted U-shaped relationship between temperature and the number of applicants. Several robustness checks and placebo tests do not alter our main findings.

Based on universities’ type and tier and climate region, we further highlight heterogeneous effects. The effect of the high temperature on the number of applicants for the UNGEE of double first-class universities that have been selected for the country’s construction plan of world-class universities and 985 universities is smaller than that of double first-class universities that have been selected for the country’s construction plan of first-class discipline and non-985 universities. In addition, the number of applicants for the universities located in cold regions is affected more by low temperature and affected less by high temperature, compared with the universities located in hot regions. Furthermore, we examine the nonlinear effects of temperature on the number of applicants by calculating temperature-bin day variables in different time spans. Our results show that temperature is an important factor affecting migration intention in both short and long periods. We also find that destination cities’ income mitigates the impacts of temperatures on migration intentions.

Talents are the core competitiveness of cities’ development both in developed and developing countries. Policymakers have formulated a series of preferential policies to increase wages and improve welfare to attract high human capital. Many researchers have proved that environmental quality and other urban amenities are becoming important factors to be considered in talents’ migration decisions. With the increasing number of extreme weather events induced by climate change, proactive measures to mitigate and adapt to climate change will be more effective for cities to attract talents. Carbon emission is one main anthropogenic cause of global climate change. Energy consumption leads to an increase in carbon emissions in the world. Both developed countries and developing countries should abide by the Paris Agreement and jointly shoulder the responsibility of mitigating climate change. The governments should reduce energy consumption, promote green and low-carbon life, and encourage low-carbon technological innovations. Our results can also help local governments effectively evaluate the social benefits of adaptation and mitigation of climate change policies.

Although this paper enriches the studies on evaluating the effects of temperature, there are shortcomings that necessitate future research. Due to data availability, we cannot further examine how the origin cities’ weather conditions affect talents’ migration intentions and identify populations whose migration intentions are more affected by temperatures.

## Figures and Tables

**Table 1:** Summary statistics.

Variable	Obs.	Mean	Std. Dev	Min	Max
**Panel A: applicants and admissions variables**, university level, 2010–2021					
Number of applicants	580	11,582.600	7467.141	237.000	41,522.000
Number of applicants (social science)	580	6690.303	5664.556	5.877	28,297.000
Number of applicants (natural science)	580	4894.095	4114.187	0.000	21,627.000
Ratio of the number of applicants and the number of admissions	580	4.214	1.736	1.150	11.786
**Panel B: temperature variables**, city level, 2009–2020					
Number of days (AT ≥ 30 °C)	284	7.239	9.512	0.000	38.000
Number of days (AT 25–30 °C)	284	35.338	20.773	0.000	90.000
Number of days (AT 20–25 °C)	284	28.701	12.955	0.000	62.000
Number of days (AT 15–20 °C)	284	13.158	11.785	0.000	67.000
Number of days (AT 10–15 °C)	284	4.866	11.684	0.000	77.000
Number of days (AT 5–10 °C)	284	0.665	2.300	0.000	22.000
Number of days (AT < 5 °C)	284	0.032	0.307	0.000	4.000
**Panel C: other meteorological variables**, city level, 2009–2020					
Precipitation (mm)	284	4.298	1.786	0.420	10.872
Relative humidity (%)	284	74.905	6.247	48.704	87.095
Wind speed (m/s)	284	2.107	0.414	1.117	3.295
Sunshine duration (hour)	284	6.174	1.272	2.487	10.309
Air pressure (0.1 hPa)	284	9641.955	735.666	6251.155	10,090.170
**Panel D: air pollution variables**, city level, 2015–2020					
PM_2.5_ ($\mu g/m^{3}$)	160	30.557	11.358	8.240	71.372
**Panel E: economics variables**, city level, 2009–2020					
Per capital GDP (RMB)	284	106,279.700	35,898.510	12,539.000	234,360.600
Average wage (RMB)	284	74,578.680	28,133.350	28,995.580	179,541.000

Notes: This table presents the summary statistics for the sample used for our main regression analysis. Observations in Panel A are at the university level from 2010 to 2021. Observations in Panel B, C, and E are at the city level from 2009 to 2020. Observations in Panel D are at the city level from 2015 to 2020.

**Table 2:** Effects of average temperature on the number of applicants.

	(1)	(2)	(3)	(4)	(5)
Number of days (≥30 °C)	−6.327	3.955	−34.907	−108.094 ***	−108.288 ***
	(31.190)	(25.071)	(28.652)	(20.694)	(28.614)
Number of days (25–30 °C)	−0.950	12.457	−4.093	−44.470 ***	−104.526 ***
	(36.127)	(31.761)	(31.774)	(1.895)	(16.136)
Number of days (15–20 °C)	64.210	40.421	62.832 **	23.596	14.321 *
	(50.388)	(31.559)	(28.476)	(19.537)	(7.253)
Number of days (10–15 °C)	28.808	27.194	44.035	−116.085 ***	11.198 ***
	(50.639)	(48.278)	(45.165)	(33.390)	(3.528)
Number of days (5–10 °C)	−45.486	−26.497	39.004	67.823	10.322
	(64.109)	(62.453)	(54.276)	(43.469)	(41.226)
Number of days (<5 °C)	−387.838 *	−386.125 **	−432.400 **	−301.501 ***	−777.355 ***
	(210.268)	(142.365)	(162.232)	(90.274)	(22.943)
Ratio (applicants/admissions)	No	Yes	Yes	Yes	Yes
Weather controls	No	No	Yes	Yes	Yes
University FE	Yes	Yes	Yes	Yes	Yes
Year FE	Yes	Yes	Yes	No	No
City-by-year FE	No	No	No	Yes	Yes
Observation	580	580	580	580	580
R^2^	0.556	0.708	0.722	0.883	0.883

Notes: The dependent variable is the number of applicants. The baseline temperature bin is 20–25 °C. Weather controls include: precipitation, relative humidity, wind speed, sunshine duration, and atmospheric pressure. Standard errors are clustered by city reported in parentheses. * *p* < 0.10, ** *p* < 0.05, *** *p* < 0.01.

**Table 3:** Effects of average temperature on the number of applicants: by discipline category.

	(1) Natural Science	(2) Social Science
Number of days (≥30 °C)	−70.956 ***	−37.198 *
	(12.394)	(20.014)
Number of days (25–30 °C)	−19.070 ***	−25.406 ***
	(1.135)	(1.833)
Number of days (15–20 °C)	20.652 *	3.001
	(11.701)	(18.895)
Number of days (10–15 °C)	−21.812	−94.177 ***
	(19.998)	(32.294)
Number of days (5–10 °C)	85.660 ***	−17.711
	(26.035)	(42.042)
Number of days (<5 °C)	−72.126	−229.114 **
	(54.069)	(87.311)
Ratio (applicants/admissions)	Yes	Yes
Weather Controls	Yes	Yes
University FE	Yes	Yes
City-by-year FE	Yes	Yes
Observation	580	580
R^2^	0.769	0.839

Notes: The dependent variable is the number of applicants. The baseline temperature bin is 20–25 °C. Weather controls include: precipitation, relative humidity, wind speed, sunshine duration, and atmospheric pressure. Standard errors are clustered by city reported in parentheses. * *p* < 0.10, ** *p* < 0.05, *** *p* < 0.01.

**Table 4:** Robustness checks: alternative specifications.

	(1) Province-Year	(2) Placebo Test
Number of days (≥30 °C)	−224.911 **	−27.802
	(91.044)	(56.223)
Number of days (25–30 °C)	−57.369	30.182
	(45.968)	(42.718)
Number of days (15–20 °C)	247.913 **	−13.519
	(97.978)	(31.539)
Number of days (10–15 °C)	508.792	4.224
	(311.789)	(41.742)
Number of days (5–10 °C)	−150.861	24.904
	(1019.337)	(38.081)
Number of days (<5 °C)	152.657	3.796
	(1549.577)	(43.327)
Ratio (applicants/admissions)	Yes	Yes
Weather Controls	Yes	Yes
University FE	Yes	Yes
City FE	Yes	No
City-by-year FE	No	Yes
Province-by-year FE	Yes	No
Observation	580	580
R^2^	0.868	0.641

Notes: The dependent variable is the number of applicants except for column (3), where the dependent variable is the log of the number of applicants. In column (1), we collapse observations by province-year. In column (2), we use the log of the number of applicants as dependent variable. Column (3) is a placebo test. The baseline temperature bin is 20–25 °C. Weather controls include: precipitation, relative humidity, wind speed, sunshine duration, and atmospheric pressure. Standard errors are reported in parentheses. ** *p* < 0.05.

**Table 5:** Robustness checks: air pollution as a possible confounder.

	(1)	(2)	(3)
Number of days (≥30 °C)	−108.094 ***	−76.885 ***	−126.825 ***
	(20.694)	(0.695)	(5.441)
Number of days (25–30 °C)	−44.470 ***	−44.304	−33.964
	(1.895)	(29.632)	(27.691)
Number of days (15–20 °C)	23.596	−208.320 ***	−190.329 ***
	(19.537)	(20.100)	(13.549)
Number of days (10–15 °C)	−116.085 ***	−60.955 ***	−64.779 ***
	(33.390)	(6.055)	(5.409)
Number of days (5–10 °C)	67.823	−68.124 ***	−114.993 ***
	(43.469)	(6.028)	(13.644)
Number of days (<5 °C)	−301.501 ***	−73.414 ***	−178.327 ***
	(90.274)	(9.492)	(63.460)
Air pollution	-	-	−173.991 ***
	-	-	(1.886)
Ratio (applicants/admissions)	Yes	Yes	Yes
Weather Controls	Yes	Yes	Yes
University FE	Yes	Yes	Yes
City-by-year FE	Yes	Yes	Yes
Observation	580	322	322
R^2^	0.883	0.858	0.858

Notes: The dependent variable is the number of applicants. Column (1) reports the baseline results from Table 2, column (4). Column (2) reports results from the same specification but only for the sample periods from 2015 to 2020. During this period, official real-time pollutant data were published. In column (3), we add controls for air pollution as measured by PM2.5. The baseline temperature bin is 20–25 °C. Weather controls include: precipitation, relative humidity, wind speed, sunshine duration, and atmospheric pressure. Standard errors are clustered by city reported in parentheses. *** *p* < 0.01.

**Table 6:** Robustness checks: alternative temperature variables.

	(1) Maximum Temperature	(2) Minimum Temperature
Number of days (≥30 °C)	−241.113 ***	−172.567 ***
	(42.239)	(20.841)
Number of days (25–30 °C)	−96.596 ***	−17.917 ***
	(4.756)	(0.837)
Number of days (15–20 °C)	37.340 ***	77.672 ***
	(0.956)	(7.801)
Number of days (10–15 °C)	−186.982 ***	−139.684 ***
	(7.090)	(3.859)
Number of days (5–10 °C)	88.671 **	120.261 ***
	(35.508)	(3.741)
Number of days (<5 °C)	−216.844 *	−226.738 ***
	(110.715)	(9.120)
Ratio (applicants/admissions)	Yes	Yes
Weather Controls	Yes	Yes
University FE	Yes	Yes
City-by-year FE	Yes	Yes
Observation	580	580
R^2^	0.857	0.857

Notes: The dependent variable is the number of applicants. The baseline temperature bin is 20–25 °C. Weather controls include: precipitation, relative humidity, wind speed, sunshine duration, and atmospheric pressure. Standard errors are clustered by city reported in parentheses. * *p* < 0.10, ** *p* < 0.05, *** *p* < 0.01.

**Table 7:** Robustness checks: alternative temperature bins.

	(1) 3 °C		(2) 6 °C
Number of days (≥30 °C)	−290.772 ***	Number of days (≥30 °C)	−322.558 ***
	(2.556)		(44.099)
Number of days (27–30 °C)	−323.770 ***	Number of days (24–30 °C)	−208.140 ***
	(6.059)		(15.519)
Number of days (24–27 °C)	−45.104	Number of days (12–18 °C)	73.994 ***
	(28.207)		(17.695)
Number of days (18–21 °C)	45.237 ***	Number of days (6–12 °C)	−119.389 ***
	(4.166)		(12.448)
Number of days (15–18 °C)	−139.365 ***	Number of days (<6 °C)	−115.680 ***
	(23.072)		(16.386)
Number of days (12–15 °C)	−55.054 **		
	(27.027)		
Number of days (9–12 °C)	−275.833 ***		
	(54.898)		
Number of days (6–9 °C)	−186.594 ***		
	(29.825)		
Number of days (<3 °C)	−248.992 ***		
	(26.592)		
Ratio (applicants/admissions)	Yes		Yes
Weather Controls	Yes		Yes
University FE	Yes		Yes
City-by-year FE	Yes		Yes
Observation	580		580
R^2^	0.857		0.883

Notes: The dependent variable is the number of applicants. The baseline temperature bin is 21–24 °C, and 18–24 °C for columns (1) and (2), respectively. Weather controls include: precipitation, relative humidity, wind speed, sunshine duration, and atmospheric pressure. Standard errors are clustered by city reported in parentheses. ** *p* < 0.05, *** *p* < 0.01.

**Table 8:** Robustness checks: alternative samples.

	(1) Beijing, Shanghai, Guangzhou	(2) Beijing, Nanjing, Shanghai, Wuhan	(3) Non “Double First-Class” university
Number of days (≥30 °C)	−179.746 ***	−117.284 ***	−81.716 ***
	(55.763)	(32.749)	(0.534)
Number of days (25–30 °C)	−93.483 ***	−12.301	−12.391 ***
	(18.169)	(28.382)	(0.882)
Number of days (15–20 °C)	37.906	137.631 ***	−70.981 ***
	(92.393)	(47.102)	(2.308)
Number of days (10–15 °C)	−89.929	38.884	−68.232 ***
	(144.264)	(73.255)	(1.168)
Number of days (5–10 °C)	87.607	69.746	−67.890 ***
	(198.892)	(121.928)	(18.610)
Number of days (<5 °C)	−411.267	−35.566	−112.022 ***
	(475.404)	(257.725)	(8.772)
Ratio (applicants/admissions)	Yes	Yes	Yes
Weather Controls	Yes	Yes	Yes
University FE	Yes	Yes	Yes
City-by-year FE	Yes	Yes	Yes
Observation	370	288	1, 092
R^2^	0.929	0.713	0.784

Notes: The dependent variable is the number of applicants. The baseline temperature bin is 20–25 °C. Weather controls include: precipitation, relative humidity, wind speed, and sunshine duration, atmospheric pressure. Standard errors are clustered by city reported in parentheses. *** *p* < 0.01.

**Table 9:** Heterogeneous effects.

	(1) Type (Comprehensive University = 1)	(2) Class (World-Class Universities = 1)	(3) Tier (985 Universities = 1)	(4) Region (North = 1)	(5) Region (Cold = 1)
Number of days (≥30 °C) × Dummy	32.039	64.482 ***	45.593 **	−61.922	113.513 **
	(42.210)	(23.534)	(20.718)	(48.421)	(42.187)
Number of days (25–30 °C) × Dummy	−43.237	55.749	4.116	−34.467	8.854 ***
	(70.476)	(40.131)	(38.542)	(50.838)	(2.560)
Number of days (15–20 °C) × Dummy	−5.671	8.065	80.929	−21.563	−70.728 ***
	(163.521)	(49.853)	(50.633)	(74.575)	(10.649)
Number of days (10–15 °C) × Dummy	364.055	70.790	51.954	237.690	−148.179 ***
	(501.996)	(86.259)	(93.718)	(264.121)	(4.243)
Number of days (5–10 °C) × Dummy	210.619 **	236.631	31.962 *	41.131	−20.164
	(99.789)	(140.866)	(16.855)	(54.827)	(38.969)
Number of days (<5 °C) × Dummy	−153.114	292.588	79.193 *	−46.389 *	−40.602 ***
	(183.290)	(522.162)	(45.452)	(22.962)	(8.699)
Ratio (applicants/admissions)	Yes	Yes	Yes	Yes	Yes
Weather Controls	Yes	Yes	Yes	Yes	Yes
University FE	Yes	Yes	Yes	Yes	Yes
City-by-year FE	Yes	Yes	Yes	Yes	Yes
Observation	580	580	580	580	580
R^2^	0.887	0.885	0.885	0.883	0.883

Notes: The dependent variable is the number of applicants. The baseline temperature bin is 20–25 °C. Weather controls include: precipitation, relative humidity, wind speed, sunshine duration, and atmospheric pressure. Standard errors are clustered by city reported in parentheses. * *p* < 0.10, ** *p* < 0.05, *** *p* < 0.01.

**Table 10:** Effects of temperature on the number of applicants in different time spans.

	(1) Past 3 Months	(2) Registration Period	(3) Past 1 Month	(4) Past 2 Months	(5) Past 6 Months	(7) Past 12 months
Number of days (≥30 °C)	−108.094 ***	−145.646	−170.010 ***	−117.861 *	−46.577 ***	−16.020 ***
	(20.694)	(119.801)	(43.813)	(60.631)	(5.876)	(0.159)
Number of days (25–30 °C)	−44.470 ***	37.646	−47.618 ***	−38.030 ***	1.529	−20.539 ***
	(1.895)	(50.257)	(1.386)	(7.018)	(1.050)	(1.765)
Number of days (15–20 °C)	23.596	−30.909	−23.452 ***	−10.692 ***	−10.995 ***	−26.462 ***
	(19.537)	(42.693)	(4.727)	(1.828)	(0.998)	(1.604)
Number of days (10–15 °C)	−116.085 ***	2.931	−4.446	−128.229 **	−13.295 ***	−33.779 ***
	(33.390)	(64.488)	(13.865)	(60.091)	(1.128)	(0.780)
Number of days (5–10 °C)	67.823	−86.310	1.757	26.178	−14.640 ***	−21.886 ***
	(43.469)	(177.093)	(1.045)	(15.827)	(1.274)	(3.502)
Number of days (<5 °C)	−301.501 ***	52.341	−221.827 ***	−222.151 ***	−167.094 ***	−120.132 ***
	(90.274)	(315.545)	(26.334)	(38.908)	(19.953)	(12.163)
Ratio (applicants/admissions)	Yes	Yes	Yes	Yes	Yes	Yes
Weather Controls	Yes	Yes	Yes	Yes	Yes	Yes
University FE	Yes	Yes	Yes	Yes	Yes	Yes
City-by-year FE	Yes	Yes	Yes	Yes	Yes	Yes
Observation	580	580	580	580	580	580
R^2^	0.883	0.857	0.883	0.883	0.857	0.857

Notes: The dependent variable is the number of applicants. The baseline temperature bin is 20–25 °C. Weather controls include: precipitation, relative humidity, wind speed, sunshine duration, and atmospheric pressure. Standard errors are clustered by city reported in parentheses. * *p* < 0.10, ** *p* < 0.05, *** *p* < 0.01.

**Table 11:** Effectiveness of mitigation: Income.

	(1) GDP per Capita	(2) Wage Payment per Capita
Number of days (≥30 °C) × Income	0.0023 ***	0.0048 ***
	(0.0000)	(0.0001)
Number of days (25–30 °C) × Income	0.0011 ***	0.0010 ***
	(0.0002)	(0.0002)
Number of days (15–20 °C) × Income	0.0032 ***	0.0012 ***
	(0.0007)	(0.0003)
Number of days (10–15 °C) × Income	0.0022 ***	0.0041 ***
	(0.0001)	(0.0002)
Number of days (5–10 °C) × Income	0.0008 ***	0.0011 ***
	(0.0000)	(0.0000)
Number of days (<5 °C) × Income	0.0017	0.0042 *
	(0.0011)	(0.0025)
Ratio (applicants/admissions)	Yes	Yes
Weather Controls	Yes	Yes
University FE	Yes	Yes
City-by-year FE	Yes	Yes
Observation	580	580
R^2^	0.883	0.883

Notes: The dependent variable is the number of applicants. The baseline temperature bin is 20–25 °C. Weather controls include: precipitation, relative humidity, wind speed, sunshine duration, and atmospheric pressure. Standard errors are clustered by city reported in parentheses. * *p* < 0.10, *** *p* < 0.01.

## Data Availability

The datasets used and analyzed during the current study are not publicly available due to privacy.

## Abbreviations

MEE: Ministry of Ecology and Environment; UNGEE: Unified National Graduate Entrance Examination.

## Appendix A. Further Discussion of the Applicant Data for the UNGEE of Double First-Class Universities

We manually collect the applicant data for the UNGEE from the official website of the graduate school of double first-class universities. First, we download annual applicant and admission data for each major from the official website of the graduate school of double first-class universities. Due to the data of some universities from many years ago has been deleted on the official website, we obtain these data from the website of the ratio of the number of applicants and the number of admissions for kaoyan (http://www.chinakaoyan.com/baolubi/ (accessed on 31 October 2021)), which summarizes annual applicant and admission data for each college from the official website of the graduate school of colleges. we have confirmed that the data from this website is consistent with the data from the official website of the graduate school. Second, we add up the total number of applicants and admissions for each school or major. Finally, we calculate the ratio of the number of applicants and the number of admissions.

## Appendix B. List of 75 “Double First-Class” Universities

**Table A1:** List of 75 “Double First-Class” Universities.

City	University
Beijing (14)	Beijing University, Renmin University of China, Beijing University of Aeronautics and Astronautics, China Agricultural University, Beijing Normal University, Minzu University of China, Central University of Finance and Economics, University of International Business and Economics, China University of Political Science and Law, Beijing University of Posts and Telecommunications, Beijing Forestry University, Capital Normal University, Beijing Foreign Studies University, University of Chinese Academy of Sciences
Shanghai (7)	Tongji University, Shanghai Jiao Tong University, East China Normal University, Donghua University, Shanghai International Studies University, Shanghai University of Finance and Economics, Shanghai University
Nanjing (5)	Nanjing University, Southeast University, Nanjing University of Posts and Telecommunications, Nanjing Forestry University, Nanjing Agricultural University
Wuhan (5)	Wuhan University, China University of Geosciences (Wuhan), Huazhong Agricultural University, Zhongnan University of Economics and Law, Central China Normal University
Guangzhou (4)	Sun Yat-sen University, South China University of Technology, Jinan University, South China Normal University
Tianjin (4)	Nankai University, Hebei University of Technology, Tianjin Medical University, Tiangong University
Xian (4)	Xidian University, Shaanxi Normal University, Northwest University, Chang’an University
Changsha (3)	Central South University, Hunan University, National University of Defense Technology
Chengdu (2)	Sichuan University, Southwestern University of Finance and Economics
Qingdao (2)	Ocean University of China, China University of Petroleum (East China)
Shenyang (2)	Northeastern University (Shenyang), Liaoning University
Chongqing (1)	Southwest University
Dalian (1)	Dalian University of Technology
Fuzhou (1)	Fuzhou University
Haikou (1)	Hainan University
Hangzhou (1)	Zhejiang University
Harbin (1)	Harbin Engineering University
Hefei (1)	Anhui University
Huhhot (1)	Inner Mongolia University
Jinan (1)	Shandong University (Jinan)
Kunming (1)	Yunnan University
Lanzhou (1)	Lanzhou University
Lhasa (1)	Tibet University
Ningbo (1)	Ningbo University
Qinhuangdao (1)	Northeastern University (Qinhuangdao)
Shihezi (1)	Shihezi University
Suzhou (1)	Soochow University
Weihai (1)	Shandong University (Weihai)
Wuxi (1)	Jiangnan University
Xiamen (1)	Xiamen University
Xianyang (1)	Northwest A&F University,
Xuzhou (1)	China University of Mining and Technology (Xuzhou)
Yinchuan (1)	Ningxia University
Zhengzhou (1)	Zhengzhou University
